# Fast quantitative urinary proteomic profiling workflow for biomarker discovery in kidney cancer

**DOI:** 10.1186/s12014-018-9220-2

**Published:** 2018-12-22

**Authors:** Lin Lin, Quan Yu, Jiaxin Zheng, Zonglong Cai, Ruijun Tian

**Affiliations:** 1 Materials Characterization and Preparation Center, Southern University of Science and Technology, Shenzhen, 518055 China; 2 Division of Advanced Manufacturing, Graduate School at Shenzhen, Tsinghua University, Shenzhen, 518055 China; 3 Department of Urology and Center of Urology, The First Affiliated Hospital of Xiamen University, Xiamen, 361003 China; 4 Department of Chemistry and Guangdong Provincial Key Laboratory of Cell Microenvironment and Disease Research, Southern University of Science and Technology, Shenzhen, 518055 China

**Keywords:** Urine, Proteome profiling, Data independent acquisition, Biomarker discovery

## Abstract

**Background:**

Urine has evolved as a promising body fluids in clinical proteomics because it can be easily and noninvasively obtained and can reflect physiological and pathological status of the human body. Many efforts have been made to characterize more urinary proteins in recent years, but few have focused on the analysis throughput and detection reproducibility. Increasing the urine proteomic profiling throughput and reproducibility is urgently needed for discovering potential biomarker in large cohorts.

**Methods:**

In this study, we developed a fast and robust workflow for streamlined urinary proteome analysis. The workflow integrate highly efficient sample preparation technique and urinary specific data-independent acquisition (DIA) approach. The performance of the workflow was systematically evaluated and the workflow was subsequently applied in a proof-of-concept urine proteome study of 21 kidney cancer (KC) patients and 22 healthy controls.

**Results:**

With this workflow, the entire sample preparation process takes less than 3 h and allows multiplexing on standard centrifuges. Without pre-fractionation, our newly developed DIA method allows quantitative analysis of ~ 1000 proteins within 80 min of MS time (~ 15 samples/day). The quantitation accuracy of the whole workflow was excellent with median CV of 9.1%. The preliminary study on KC identified 125 significantly changed proteins.

**Conclusions:**

The result suggested the feasibility of applying the high throughput workflow in extensive urinary proteome profiling and clinical relevant biomarker discovery.

**Electronic supplementary material:**

The online version of this article (10.1186/s12014-018-9220-2) contains supplementary material, which is available to authorized users.

## Background

Kidney cancer (KC) accounts for about 3% of all adult cancers and is one of the most fatal diseases of the genitourinary system [1]. Early diagnosis of KC is crucial for the patients as it associates with excellent 5-year survival rate. However, around 30% of KC patients are diagnosed at the metastatic stage and are characterized with poor prognosis [2, 3]. The pathogenesis of KC have not been fully elucidated and there is still no acknowledged biomarker which can be used for its diagnosis or prognosis [4]. Discovery of KC related potential biomarkers has important clinical significance.

Urine is an ideal detection objective for identifying biomarkers of urological malignancies as it can be easily and non-invasively obtained and contains cells and proteins that originate from urogenital system [5, 6]. Furthermore, as a glomerular filtrate of plasma, the urine proteome can reflect physiological and pathological status of the human body [7]. Recent studies show that urinary proteome has great potential in classification and diagnosis both urogenital and systemic diseases [8–14]. However, urine is also a difficult proteomic sample to work with, due to its low protein concentration, high dynamic range of protein expression and high inter-individual variability. Compared with human plasma and tissue proteome, the urinary proteome has been relatively less studied.

Mass spectrometry (MS) has evolved as the mainstay for high-throughput proteome profiling of complex biological samples recently. Many efforts have been made to characterize urinary proteomes using MS in recent years. The regular urinary sample preparation process usually include protein extracted by organic solvent precipitation, protein re-dissolving and overnight digestion. Furthermore, to increase the detection depth, protein or peptide fractionation prior to LC–MS-analysis is currently widely employed. These processes typically include gel electrophoresis, ion exchange chromatography or high-PH reversed phase (RP) chromatography [15, 16]. Using such strategies, it has now become possible to identify more than 1500 proteins in a single urine sample [17] and even more than 6000 proteins when using hundreds of fractions from multi-dimensional separation strategies and pooled urine from dozens of humans [18]. However, the pre-fractionation steps are undesirable in clinical application because they raise pre-analytical perturbations and restrict throughput. Due to the exertive processes of sample preparation and limited throughput, small sample size is usually enrolled in the discovery phase of current urinary biomarker studies, which reduces the credibility of the discovered biomarkers [19]. Up to now, few urinary biomarkers derived from ‘discovery’ studies have been successfully translated into clinical practice [20]. Increasing the urine proteomic detection throughput and reproducibility is urgently needed for discovering and verifying biomarkers with large sample cohorts.

A high-throughput proteome profiling workflow should include both efficient sample preparation method as well as robust MS acquisition approach. For sample preparation, the development of integrated proteomics sample preparation technologies has attracted increasing attention recently. Mann’s group reported an in-StageTip approach for quickly processing of cell and plasma samples in less than 2 h [21, 22]. Recently, a simple and integrated spintip-based proteomic technology (SISPROT) was developed by our group and has been successfully used in proteomic profiling of cell [23], tissue [24], microbiome [25], and plasma [26]. It could be of great significance to apply such technologies for urinary sample preparation. In terms of MS analysis, data-dependent acquisition (DDA) is remaining the most widely adopted MS approach for untargeted screening in discovery proteomics. However, the semi-stochasticity of precursor ion selection and non-uniformity of scan point leads to poor reproducibility and compromise the accuracy of quantitative analysis [27, 28]. Compared with DDA, data-independent acquisition (DIA) method is emerging as a powerful technology for quantitative proteomics recently [29]. The DIA approach divides all the precursor ions into several consecutive windows for fragmentation, and acquires all the resulting fragments in each isolation window. It features with high-throughput, quantitative consistency, and traceable data, which is very suitable for proteomic biomarker discovery studies [30, 31].

**Fig. 1:**
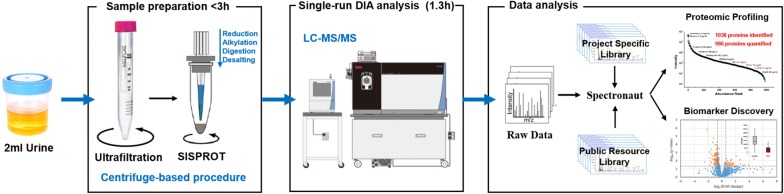
Workflow of the high-throughput urinary proteome profiling

In this paper, a streamlined urinary proteome profiling platform was developed by integrating highly efficient sample preparation procedure and urinary specific variable window DIA approach (Fig. 1). Starting with 2 mL of urine, all the preparation processes were performed in a centrifuge-based procedure within 3 h and can be easily multiplexed. Without pre-fractionation, our developed DIA approach enables the quantification of ~ 1000 proteins with 80 min gradient time on an Orbitrap Fusion mass spectrometer. Furthermore, we compared the performance of the project specific library with two resource libraries. The results indicate the great potential of using resource spectral libraries for DIA data analysis. The developed workflow was then applied in a proof of concept urinary proteome study of KC.

## Methods

### Sample collection

The first-morning urine samples of 21 KC patients and 22 healthy controls were collected from First Hospital of Xiamen. The KC patients were diagnosed by histopathological examination and none had undergone nephrectomy before sample collection. Samples with hematuria or proteinuria were excluded from the study. The clinical information of the KC patients is provided in Additional file 1: Table S1. Samples were centrifuged at 2000×*g* for 10 min at 4 °C to sediment cellular fragments and then stored at − 80 °C before analysis. For construction of project specific spectral library, two pooled urinary samples were prepared by mixing equal volume of each sample in each condition.

### Urine sample preparation

Urine samples were prepared with a centrifuge-based protocol, which combined ultrafiltration and recently developed SISPROT technology [23] with optimization for urine. 2 mL neat urine were concentrated using an ultrafiltration devices (Amicon^®^ Ultra-4 10 K, Merck) according to the instructions. The protein concentration of each urine were measured using Bradford protein assay (Bio-Rad, USA). The brief procedure for SISPROT is as following. Firstly, the SISPROT tip was made by filling 5 pieces of C18 disk (3 M Empore, USA) and then 2 mg POROS SCX beads (Applied Biosystems, USA) into a standard 200 μL pipette tip. Secondly, the urine sample (~ 20 μg of protein) was acidified with 0.1% (v/v) formic acid to pH 2–3 before loading onto the SISPROT tip by centrifugation. After washing with 20% (v/v) acetonitrile (ACN) in 8 mM potassium citrate buffer (pH 3), the proteins were then reduced with 10 mM Tris (2-carboxyethyl) phosphine hydrochloride (TCEP) in 9 mM potassium citrate buffer (pH 3) for 15 min (at room temperature). Protein digestion was subsequently carried out by injecting into the tip with 2 μg/μL trypsin (Promega, Madison, WI) in 10 mM iodoacetamide (IAA), 100 mM Tris–HCl (pH 8), and incubating in darkness for 1 h (at room temperature). The peptides were subsequently transferred to the C18 disk with 200 mM ammonium formate (AF, pH 10). After washing with 5 mM AF (pH 10), the peptides were directly eluted by 40 μL of 80% (v/v) ACN in 5 mM AF (pH 10) or fractionated by a stepwise increasing gradient of ACN (3, 6, 9, 15, and 80%) in 5 mM AF (pH 10). Finally, the obtained peptides were dried and resuspended in 10 μL of 0.1% (v/v) formic acid with iRT peptides (Biognosys, Schlieren, Switzerland) adding into each sample according to manufacturer’s instructions for LC–MS analysis. The entire sample preparation process takes less than 3 h and can be multiplexed on centrifuges with excellent reproducibility.

### DDA acquisition and data analysis

DDA analysis was performed on an Orbitrap Fusion mass spectrometer connected to an EASY-nLC 1000 system (Thermo Fisher Scientific). The peptide separation was performed on an integrated spray-tip analytical column (75 μm i.d. × 20 cm) packed with 1.9 μm ReproSil-Pur 120 Å C18 resins (Dr. Maisch GmbH, Ammerbuch, Germany). A binary buffer system of 0.1% (v/v) formic acid in water (buffer A) and 0.1% (v/v) formic acid in ACN (buffer B) was used for separation at a flow rate of 250 nL/min. The injection volume is 2 μL. A 80 min gradient was performed as follows: from 3 to 7% B in 2 min, from 7 to 22% B for 50 min, from 22 to 35% B for 10 min, from 35 to 90% B in 2 min, and held at 90% B for 16 min. The DDA method consisted of a full MS scan over m/z range of 350–1550 at a resolution of 120,000 in the Orbitrap mass analyzer followed by data dependent MS/MS scans with a Top Speed method (3 s). MS/MS was carried out in the Orbitrap mass analyzer with a resolution of 15,000 using an isolation window of 1.6 *m*/*z* and HCD fragmentation with normalized collision energy (NCE) of 30%. The dynamic exclusion time was set to 40 s.

The Sequest HT [32] node integrated within the Proteome Discoverer (PD) software (Version 2.1, Thermo Fisher Scientific) was applied to search the raw data against the human Uniprot fasta database (70,947 entries, downloaded on Mar 10, 2017) appended with the Biognosys iRT peptides sequence. A maximum missed cleavages of two were allowed. Carbamidomethylation of cysteine was chosen as static modification, while oxidation of methionine and deamidation of asparagine and glutamine were set as dynamic modifications. False discovery rate (FDR) was set to 1% for peptide spectrum matches and proteins. MaxQuant [33] (version 1.5.2.8) was applied for the label-free quantification (LFQ) analysis of proteins with default settings. The same protein sequence database and same modification parameters were used as the PD search. The FDR was controlled as 1% for both peptide spectrum matches and proteins.

### Generation of project specific library

For generation of project specific library, the pooled urine samples were measured in two ways. The samples were fractionated into 5 fractions with high-PH RP fractionation strategy and all the fractions were measured by DDA. Unfractionated samples were analyzed with DDA in four technical replicates. The injection volume is 2 μL for unfractionated sample and 5μL for fractionated components. The acquired DDA data were searched with PD software and the search result was imported to Spectronaut 11.0 (Biognosys) for library generation. The default settings were used as follows: fragment ions were selected over *m*/*z* range of 300 to 1800, fragments per peptide were restricted from 3 to 6, and a FDR threshold was set as 0.01.

### DIA sample acquisition and data analysis

The DIA experiments were carried out on the same instrument as DDA. The same LC separation conditions were applied. A variable window DIA approach (termed as vDIA) specific for urine samples was developed. The precursor *m*/*z* distribution was obtained according to the DDA experiment of the pooled urine samples and the window list was established based on the criteria of equalizing the number of parent ions in each isolation window [34]. Each DIA cycle consisted of a full MS scan over *m*/*z* range of 350–1400 with a resolution of 60,000 in the Orbitrap mass analyzer followed by 30 vDIA scans with a resolution of 30,000 and NCE of 30%. The cycle time was 2.4 s. The classic DIA approach contains a full MS scan and 32 DIA scans with fixed isolation width. The full scan was set over *m*/*z* range of 395–1205 with a resolution of 60,000; followed by DIA scans with 26 m/z fixed isolation width, resolution of 30,000 and NCE of 30% [35]. Details of the two methods are listed in Additional file 2: Table S2 and Additional file 3: Table S3.

DIA data were analyzed with Spectronaut 11.0 (Biognosys) with default settings as follows: peak detection, dynamic iRT; correction factor, 1; interference correction on MS2 level, enabled; and cross run normalization, enabled. Protein inference was performed with the ID picker algorithm [36] in Spectronaut. The FDR cutoff was set as 1% for both peptide and protein levels.

For statistical analysis, the data were further analyzed by SPSS (version 18.0). Student’s *t* test was used to calculate the significance of protein intensity changes. Gene ontology (GO) enrichment was performed using DAVID (version 6.8) [37, 38].

## Results

### Development of urinary sample preparation procedure

In this study, we tried to apply the SISPROT technology for high efficient urine sample preparation. Due to the relatively low protein concentration feature of urine sample and the limited loading volume of SISPROT device, it is impractical to use SISPROT technology to process urine sample directly. As an attempt, ultrafiltration technology was combined with SISPROT for sample preparation in our study. The neat urine were rapidly concentrated and desalted by ultrafiltration device. After protein concentration measurement, ~ 20 ug of protein filtrate were used for subsequent protein digestion on SISPROT. The digestion efficiency of urine sample on SISPROT was evaluated by calculating the missed cleavage rates of our DDA data by performing an additional search in the PD software allowing for maximum missed cleavages of ≤ 5. As shown in the Additional file 4: Table S4, ~ 99% of PSMs have a missed cleavage number of ≤ 2, which indicates the high efficiency of the SISPROT technology in processing urine samples. The entire sample preparation process takes less than 3 h and allows multiplexing on standard centrifuges.

### Development of urine specific DIA method

**Fig. 2:**
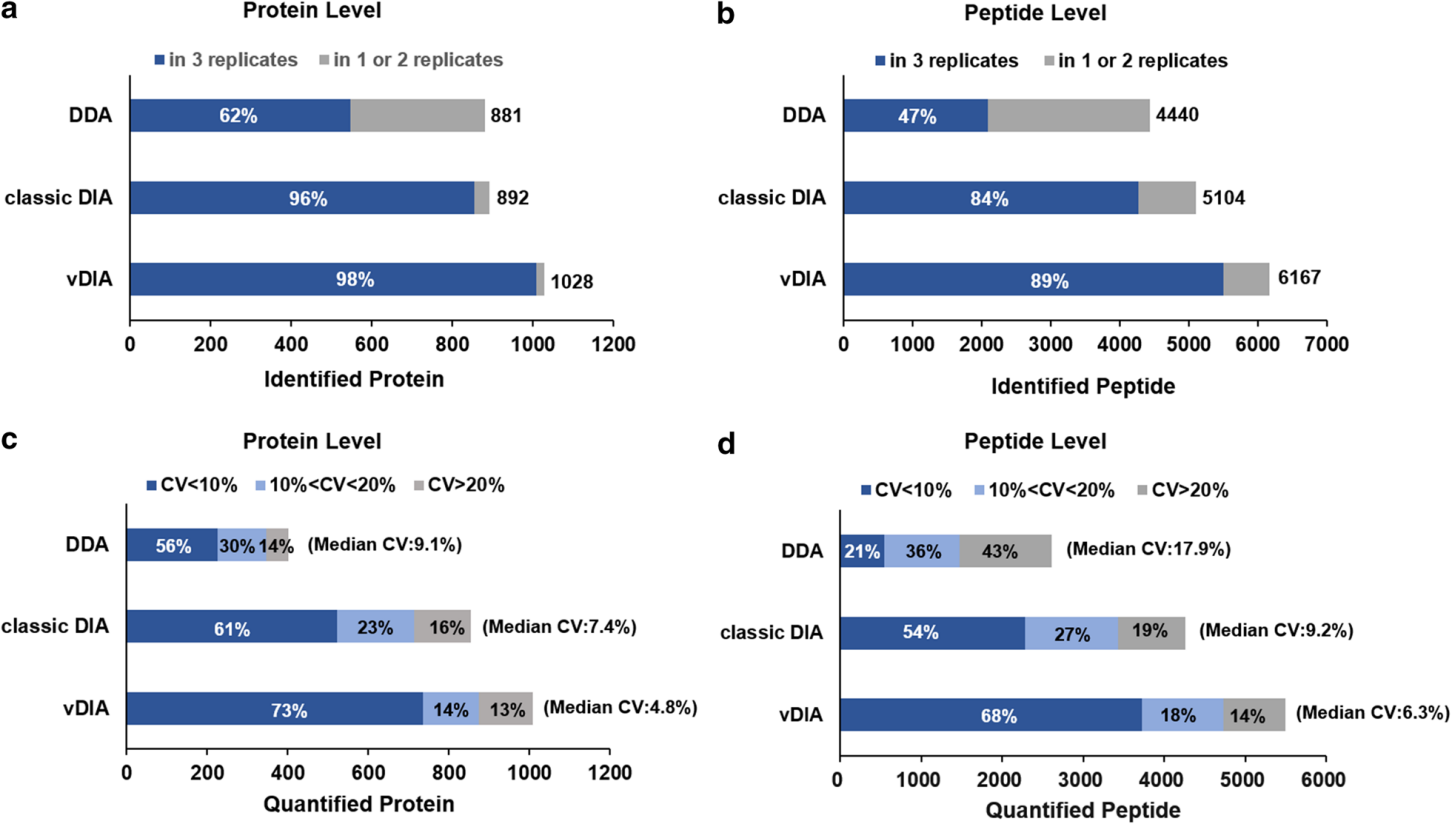
Performance comparison among standard DDA, classic DIA and vDIA over three repeated acquisitions. **a**, **b** Total number of identified proteins and peptides were plotted. The column is separated into proteins or peptides which were detected in common (dark blue) and those detected in only one or two replicates (gray). **c**, **d** Distribution of quantified proteins and peptides in each CV% levels

The regular DIA method separate the whole mass range into several fixed isolation windowes. Due to the sample specific and uneven precursor ions distribution over the mass range, the number of precursor ion in each isolation window varies greatly when using fixed window width. If the distribution of parent ions in each isolation window is averaged, the detection performance will be improved. Varesio et al. found that variable windows in SWATH-MS acquisition can improve selectivity in proteomic analysis of cell sample [34]. As an attempt, we try to develop a variable window DIA (vDIA) approach that is specifically targeted for urine samples here. The precursor m/z distribution of the urine sample was firstly obtained based on the DDA mode experiment on the pooled urine samples, and the variable window lists were then constructed based on the criteria of equalizing the number of precursor ions per each isolation window. To investigate how the method performs when comparing with standard DDA and classic DIA, the same urine sample was measured over three replicates by the three methods, respectively. As shown in Fig. 2, the methods were benchmarked based on the number of measured peptides and proteins, identification reproducibility and quantitation accuracy.

The reproducibility was evaluated by calculating the percentage of peptides and proteins that identified in common over three repeated acquisitions. For DDA method, 881 proteins and 4440 peptides were identified in total, and only 547 (62%) proteins and 2095 (47%) peptides identified in common cover three replicates. The identification reproducibility was poor due to the semi-stochastic nature of DDA and may also due to the high protein diversity in both abundance and composition of urine sample. The data reproducibility was greatly improved in both protein and peptide levels when using DIA methods. Comparing with classic DIA approach, the number of proteins and peptides identified by vDIA method have increased 15.2% and 20.8%, respectively, with improved reproducibility. The identification reproducibility is as high as 98% in protein level with our developed vDIA method.

In order to compare quantitative performance, the DDA data were analyzed using MaxQuant for label-free quantification (Fig. 2c, d) [33]. As a result, 402 proteins were quantified with a median coefficients of variation (CV) of 9.1% for DDA. For classic DIA and vDIA methods, 855 proteins and 1008 proteins were quantified, respectively. Compared to the standard DDA method, the vDIA method quantified 2.1 times peptides and 2.5 times proteins with a much better quantitative precision. It is worth mentioning that median CV% of our vDIA method in protein quantification is as low as 4.8%, with 73% of proteins showing CVs of ≤ 10% and 87% of proteins showing CVs of ≤ 20%.

### Use of resource libraries for analysis of DIA data

**Fig. 3:**
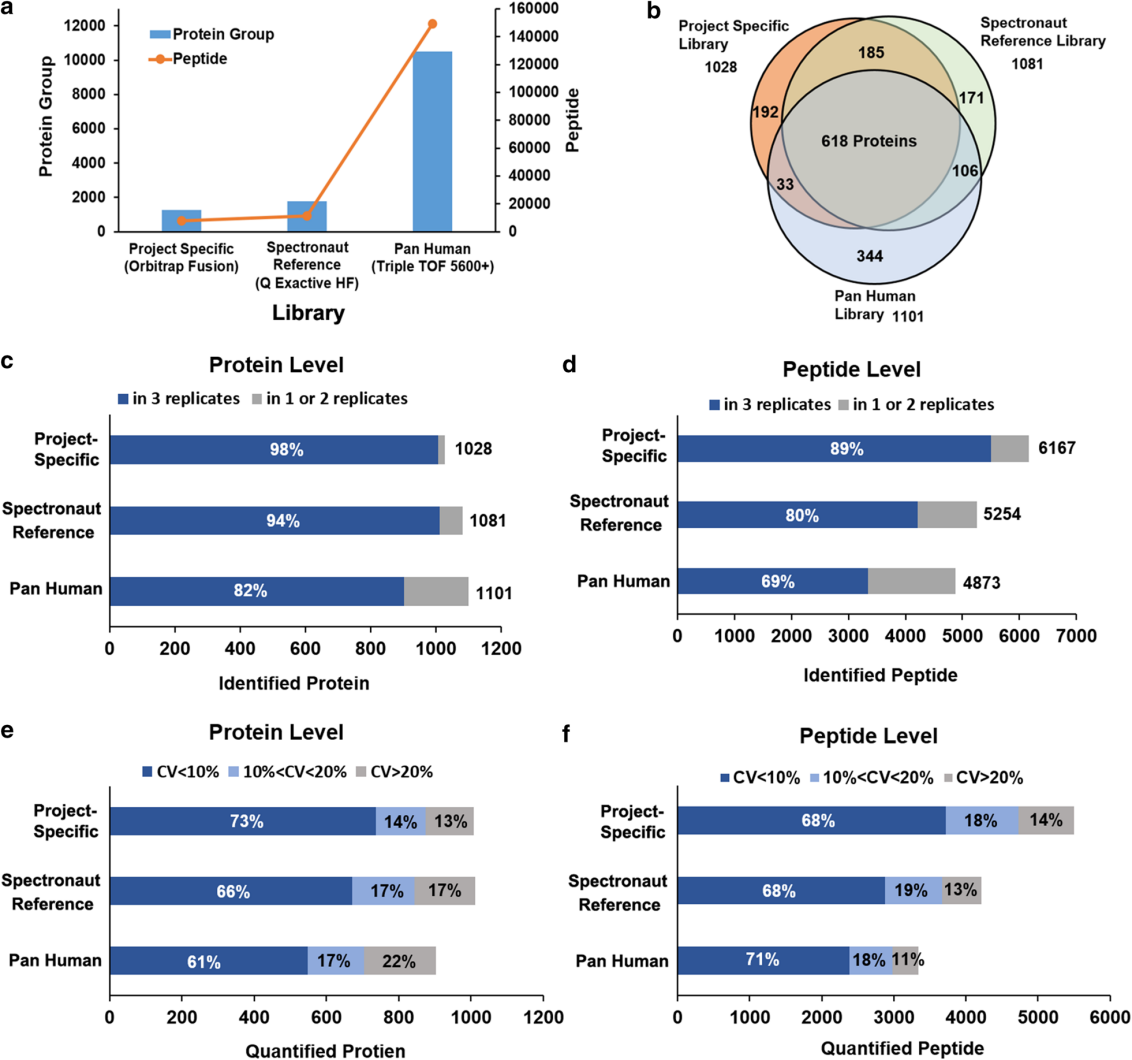
Comparison between DIA spectral libraries. The analysis of the vDIA data was carried out with three spectral libraries: a project specific, a urine reference library appended in Spectronaut and the pan human library. **a** Basic information of the spectral libraries: the number of proteins and peptides covered by the libraries and the MS instrument used for library generation. **b** Overlap of identified proteins depending on the spectral libraries. **c**, **d** Total number of identified proteins and peptides were plotted. The column is separated into proteins or peptides which were detected in common (dark blue) and those detected in only one or two of three replicates (gray). **e**, **f** Distribution of quantified proteins and peptides in each CV% levels

To construct high-quality spectral library that contains MS coordinate information for targeted data extraction is critical for DIA data analysis. Generation of internal and project specific library is currently preferred method for analyzing of DIA data. Although there are some public available resources, the differences in separation gradients and instruments have limit the usage of these libraries [31]. However, recent improvement in retention time prediction approaches have improve such problems and indicate the possibility of using such external spectral libraries [30, 39]. In this study, two resource libraries were tested for targeted analysis of our vDIA data and the performance of the libraries were compared with internally built and project specific library. The basic information of the libraries is summarized in Fig. 3a (More detailed information is provided in Additional file 5: Table S5). Spectronaut reference library is a Biognosys online urine reference library appended in the Spectronaut software, which was built based on 14 DDA acquisitions (2 h gradient) of fractionated urine samples. As shown in Fig. 3c, d, 1081 proteins and 5254 peptides were identified with the library. Although fewer peptides were identified than the project specific library, the number of identified proteins was comparable. The identification reproducibility of three replicates was 94% for proteins and 80% for peptides. For quantitative precision, 1012 proteins were quantified with a median CV of 5.9%. Although the reproducibility and quantitative precision is still not as good as the project specific library, the performance is good enough for practical application. In addition, 803 proteins (74% of all) detected by the library were overlap with the project specific library (Fig. 3b), indicating the high relevance of the two libraries and the accuracy in protein detection. To further assess the effect with regard to the source of libraries, a pan human library published by Rosenberger et al. was applied [40]. The library was built with a different instrument type, a time of flight (TOF)-MS, and was generated based on 331 DDA measurements from fractionated samples of different human cell lines, tissues and affinity enriched protein samples, which covers more than 10,000 human proteins. As shown in Fig. 3c–f, the performance of this large repository human library is inferior to the Spectronaut urine reference library. Overly large spectral libraries that only a fraction can be recovered from the data might reduce the sensitivity and peptide assignment confidence of the targeted analysis. The lower identification reproducibility with this library was probably due to the irrelevant increase in large search space and variability of instrument dependent peptide fragmentation. However, 903 proteins were still quantified with a small median CV of 6.7%, which is much better than regular DDA analysis. All the results indicate the great potential of using resource spectral libraries for DIA data analysis. Usage of public available resource libraries will save great amount of MS time for generating internal specific library, which could be very attractive in future DIA data analysis.

### Performance of the fast profiling workflow

**Fig. 4:**
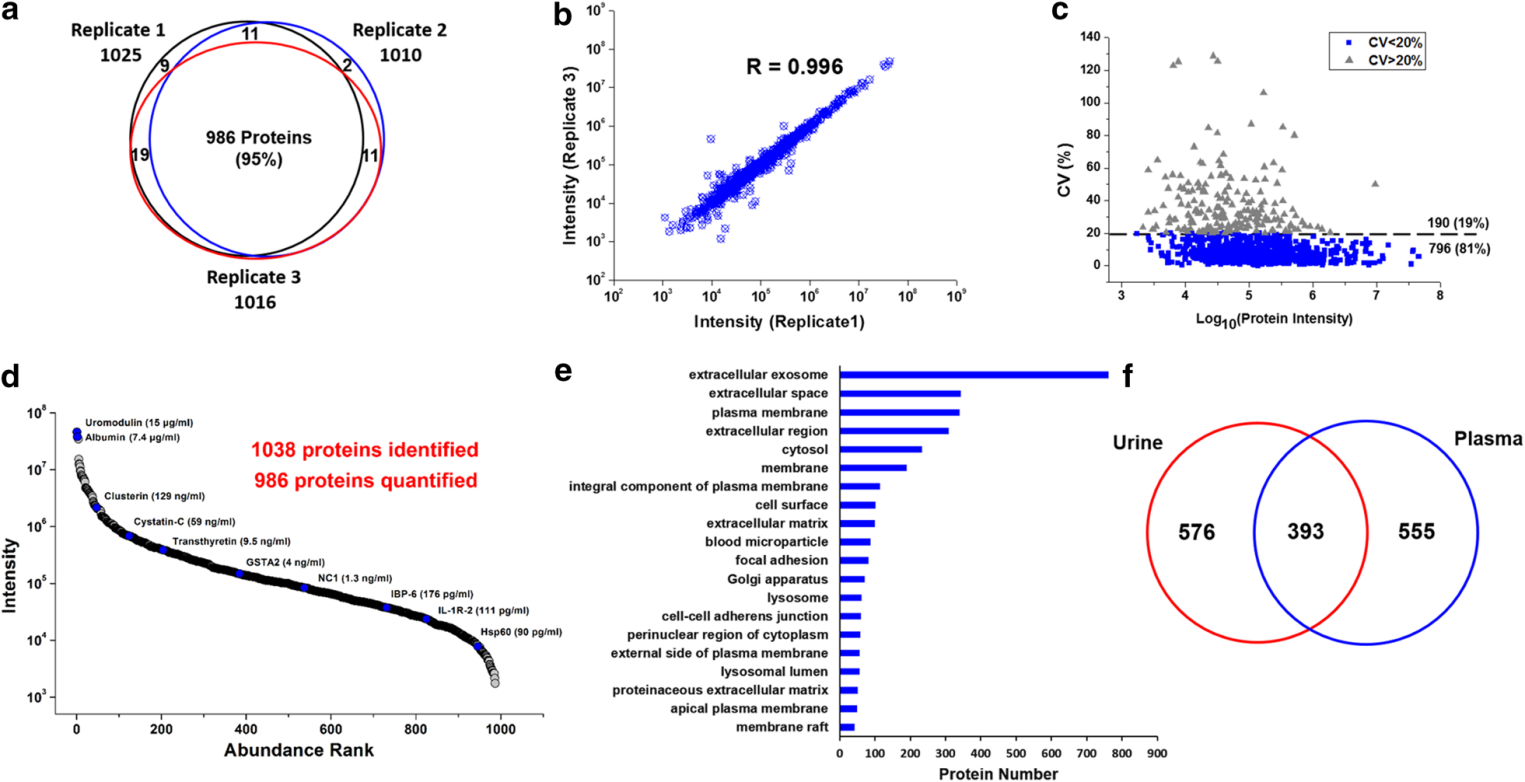
Performance of the urinary proteome profiling workflow. **a** Identification reproducibility of the entire workflow. **b** Correlation of the quantified protein intensities between replicate 1 and 3. Correlation of the protein intensities between replicate 1 and 2, and replicate 2 and 3 were appended in Additional file 4: Figure S1. **c** Quantification precision of the workflow. **d** Abundance distribution of the quantified proteins. Some disease-related biomarkers with known urinary concentration as reported form previous immunoassay screening are labeled [41]. *GSTA2* Glutathione S-transferase A2, *NC1* Collagen alpha-1(XVIII) chain, *IBP-6* Insulin-like growth factor-binding protein 6, *IL-1R-2* Interleukin-1 receptor type 2, *Hsp60* 60 kDa heat shock protein. **e** Top 20 of Gene Ontology enrichments for cellular component. **f** Comparative analysis of the urine and plasma proteome. The protein identifiers in different datasets were all converted to gene names for easier comparison

The performance of the whole workflow including sample preparation and MS analysis was evaluated by analysis of three batches of a same urine samples. As show in Fig. 4a, 1025, 1010 and 1016 proteins were identified from each replicate, with 986 (95%) proteins identified in common. Figure 4b and Additional file 6: Figure S1 show the response correlation of the quantified proteins between individual replicates. The average correlation coefficient was 0.992, suggesting that our workflow has a good quantification precision (The result in peptide level are appended in Additional file 7: Figure S2). CV of protein intensity of three replicates were calculated and the median CV% was 9.1%, with 81% of proteins showing CVs of ≤ 20% (Fig. 4c).

Our integrated workflow enables reproducibly quantification of 986 proteins with a 80 min single-shot DIA analysis. As illustrated in Fig. 4d, the dynamic range of quantified protein intensity cover nearly five orders of magnitude. Among them, uromodulin and albumin were the two most abundant proteins, and PERG-1 (Retinoic acid receptor responder protein 1) was the least abundant one (Additional file 8: Table S6). The concentrations of some of the proteins were reported in the tens of pg/mL level, such as Interleukin-1 receptor type 2 (110 pg/mL), 60 kDa heat shock protein (90 pg/mL) and fractalkine (40 pg/mL) [41].

GO analysis was performed to provide insight into the cellular component of the quantified proteins. As illustrated in Fig. 4e, GO terms related to extracellular proteins (such as extracellular exosome, extracellular space and extracellular region), plasma membrane and cytosol were most overrepresented, which was consistent with recent large-scale urinary proteomic studies [18, 42, 43]. As urine is a glomerular filtrate of plasma, we compared our urinary proteome data with a recently reported plasma proteome data to check how many plasma-related proteins could be detected in urine. The plasma proteome dataset has a comparable detection depth of 1040 proteins and was reported by Mann’s group through extensive peptide fractionation and 16 h of MS time [22]. As shown in Fig. 4f, a total of 393 (41.5%) of the proteins identified in the deep plasma proteome were common to the proteins quantified by our fast urinary proteome profiling (40.6%). This result was largely consistent with previous report that approximately one-third of urinary proteins originate from the plasma proteins [44]. We further searched for the presence of approved blood biomarkers in our urinary proteome dataset. Among the 109 Food and Drug Administration (FDA) approved blood biomarkers [45], 53 (~ 50%) of them were reproducibly quantified in our list (Additional file 8: Table S6). As urine can be obtained in a more convenient and non-invasive way compared with blood, it would be significant if these blood biomarkers could be confirmed as urinary biomarkers. In addition, as a biofluid closest to kidney, urine was thought to contain rich information which could reflect kidney function and an ideal source for discovering kidney disease biomarkers. Lately, Gao’s group reviewed 38 reported urinary candidate biomarkers of glomerular and tubular injury [18]. As indicated in Table S4, 25 of those biomarkers can be detected in our list. All the above results indicate the great potential of our fast urinary proteomic profiling in disease relevant biomarker detection.

### High-throughput urinary proteome profiling of KC

**Fig. 5:**
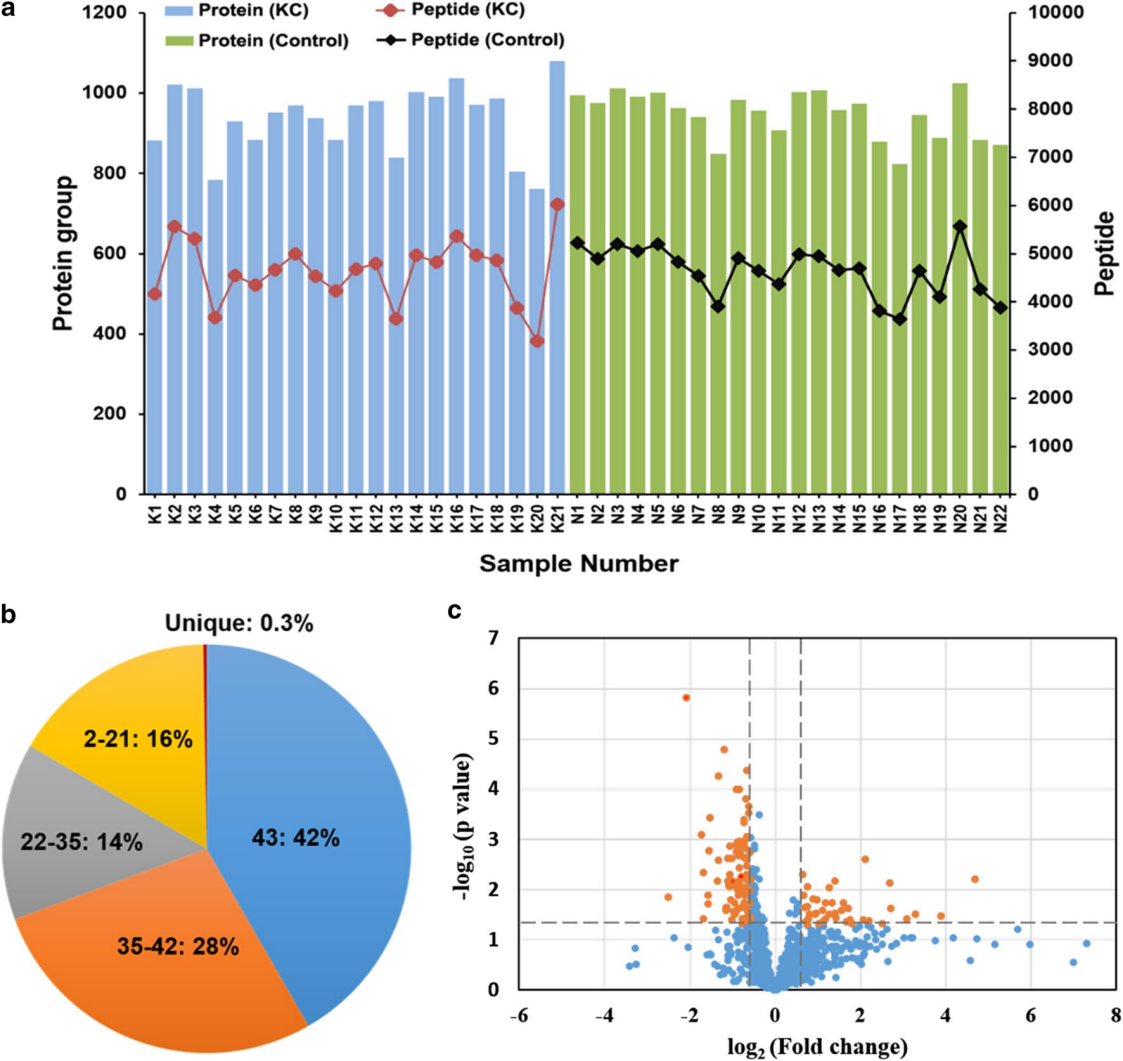
High-throughput urinary proteome profiling of KC. **a** Number of detected proteins and peptides in each urinary sample. **b** Percentage of detected proteins in all the 43 samples, in 35–42, in 22–35, in 2–21 samples or in only one sample. **c** Volcano plot of protein abundance changes between KC patients and controls. Significantly changed proteins are labelled in orange

After establishing the optimized workflow, we applied it to a urine proteomic study of KC. Twenty-one KC patients and 22 healthy controls were involved in the study. After processed using our centrifuge-based approach, a total of 43 samples were measured by our urine specific DIA method within 3 days. An average of 942 protein groups and 4632 peptides per sample (Fig. 5a) were detected, and 1163 protein groups and 7361 peptides were detected in total from 43 samples. We further calculated the frequency of protein detection (Fig. 5b). 485 proteins (~ 42% of the all) were detected in common in all the 43 samples, 807 proteins (~ 70%) in most (> 80%) of the samples, and only 3 proteins (0.3%) detected individually in one sample. The result indicated the accepted stability and reproducibility of our workflow in large scale urine proteome profiling. Student’s *t* test was applied to detect the disease related potential markers. As indicated in the volcano plot (Fig. 5c), 125 proteins were found differentially expressed between KC patients and controls (p value < 0.05 and Fold change > 1.5). Among them, 85 proteins were significantly down-regulated in KC patients, while 40 proteins were significantly up-regulated. Detailed information of these changed proteins is provided in the Additional file 9: Table S7.

## Discussion

Many efforts have been made to characterize more urinary proteins in recent years, but few have focused on the analysis throughput and detection reproducibility. In this study, we aim to develop a fast and robust urinary proteome profiling platform that can be applied in large-scale clinical detection. We reasoned that the entire process of such a platform, including sample preparation, MS acquisition and data analysis, ought to be fast, reproducible and convenient. In this regard, sample preparation processes were tried to be simplified and pre-fractionation procedures were overleaped. According to the characteristics of urine samples, a centrifuge-based protocol, which combined ultrafiltration and the SISPROT technology, was developed for urine sample processing. Starting with 2 mL of neat urine, the entire sample preparation process takes less than 3 h and allows multiplexing on standard centrifuges. For MS analysis, a urine specific vDIA method of 80 min gradient was developed (~ 15 samples per day). The acquired DIA data was subsequently analyzed with commercial available Spectronaut software. The whole workflow takes less than 5 h (Fig. 1). The performance of the whole workflow was evaluated, and both the detection reproducibility and quantitative precision were found excellent. It is noteworthy that the quantification precision of a < 10% median CV of the workflow is much smaller than the biological CV (reported as 60–70%) [46], which allows us to omit technical replicates for both sample preparation and MS acquisition, and to focus on other biological samples, which greatly improves the analysis throughput.

The high throughput workflow allows quantitative analysis of ~ 1000 proteins in single urine sample, which covers low abundance urinary proteins with reported concentration in the pg/mL level. It is worth mentioning that, comparing with plasma, urine is easier to be profiled to a relatively deep protein depth. With our study, over one thousand proteins can be detected with 80 min of MS time. But for plasma proteome, due to the negative effects of high abundance blood proteins, a comparable detection depth can only be obtained through extensive peptide fractionation and consuming more than 10 h of MS time. It could be a distinct advantage of urine over blood as a non-invasive biofluid in future clinical proteomics.

The workflow was further applied in a proof-of-concept urine proteome study of KC. During the analysis of a total of 43 samples, the workflow presented satisfactory stability and reproducibility in protein detection. 125 proteins were found differentially expressed between the KC patients and healthy controls. Some of the proteins have been detected as potential biomarkers for KC or associated with tumor growth and proliferation in previous studies, such as Multimerin-2 [47, 48], transmembrane protein 106A [49, 50], apolipoprotein D [51], vasorin [52], ras-related protein Rab-14 [53], retinol-binding proteins 5 [54], neuronal growth regulator 1 [55] and prolactin-inducible protein [56, 57].

However, these significantly changed proteins can’t yet be treated as potential biomarkers for KC in current preliminary study. Due to the unique characteristics of urine, such as high inter-individual variability and susceptibility to various condition of the urinary system, larger sample cohorts and patients with non-malignant kidney disease controls are needed in a comprehensive discovery study to obtain more conclusive result. Another problem with current MS-based biomarker discovery studies on urine is that there is a lack of consistency in normalization of protein concentration. In this study, to correct for the different dilution factors among urine samples, same protein amount were used for digestion for different samples. The potential problem with this widely used total protein normalization strategy is that samples with proteinuria or hematuria could weaken the differences in biomarker levels between the patients and controls [58]. Urinary creatinine-based normalization is another widely applied strategy. The potential problem with the strategy is that the excretion of creatinine is susceptible to renal function, muscle mass and metabolism, and can be dependent on exogenous factors such as age, gender and mental state [59]. Further studies needed to be carried out to compare those normalization methods. Due to the limit of current sample size and significantly more effort for developing commonly accepted normalization method, such comprehensive study on KC is out of the scope of this study and will be carried out in our future study. However, the current proof-of-concept study suggested the feasibility of our workflow in extensive urine proteome profiling and clinical relevant biomarker discovery.

## Conclusions

In this study, we develop a fast and robust workflow for streamlined urinary proteome analysis. The workflow integrate highly efficient centrifuge-based sample preparation technology and urinary specific DIA approach, that enables reproducibly quantification of ~ 1000 proteins with 80 min MS time. Compared to the standard DDA strategy, our DIA method quantified 2.1 times peptides and 2.5 times proteins with better quantitation precision. In addition, we evaluated the feasibility of using resource libraries for DIA data analysis. Although the overall performance of the public resource libraries is still not as good as that of project specific library, they present great potential to be used in future data analysis. The workflow was subsequently applied to a urine proteomic study of KC. A total of 43 samples were analyzed by the integrated workflow within 3 days. 125 proteins were found differentially expressed in KC patients. The result suggested the feasibility of applying the workflow in extensive urinary proteome profiling and clinical relevant biomarker discovery.

## Additional files

**Additional file 1: Table S1.** Clinical information of the KC patients.

**Additional file 2: Table S2.** A summary of the vDIA method.

**Additional file 3: Table S3.** A summary of the classic DIA method.

**Additional file 4: Table S4.** Digestion efficiency of the SISPROT technology in processing urine samples.

**Additional file 5: Table S5.** Overview of the spectral libraries.

**Additional file 6: Figure S1.** Correlation of the quantified protein intensities between (a) replicate 1 and 2, and (b) replicate 2 and 3.

**Additional file 7: Figure S2.** Performance of the urinary proteome profiling workflow in peptide level. (a) Peptide identification reproducibility of the entire workflow. (b–d) Correlation of the peptide intensities between individual replicates.

**Additional file 8: Table S6.** The quantified urinary protein list.

**Additional file 9: Table S7.** Significantly changed proteins found in KC patients.

## Abbreviations

DIA: data-independent acquisition
KC: kidney cancer
MS: mass spectrometry
RP: reversed phase
SISPROT: a simple and integrated spintip-based proteomic technology
DDA: data-dependent acquisition
IAA: iodoacetamide
AF: ammonium formate
NCE: normalized collision energy
PD: Proteome Discoverer
FDR: false discovery rate
LFQ: label-free quantification
GO: gene ontology
vDIA: variable window DIA
PERG-1: retinoic acid receptor responder protein 1
FDA: Food and Drug Administration
