# Supplementary material for: Fast quantitative urinary proteomic profiling workflow for biomarker discovery in kidney cancer
Source: Clin Proteomics. 2018 Dec 22;15:42. doi: 10.1186/s12014-018-9220-2 (PMC6303996; doi:10.1186/s12014-018-9220-2)
Supplement: Supplementary file 1 — Additional file 1: Table S1. Clinical information of the KC patients. [file 12014_2018_9220_MOESM1_ESM.docx]

**Table S1.** Clinical information of the KC patients.

| Characteristics | KC patients | |
| --- | --- | --- |
| Age | Mean | 57.3 |
|  | Range | 34-77 |
| Gender | Male | 9 |
|  | Female | 12 |
| Cancer stage | T1a | 12 |
|  | T1b | 7 |
|  | T2 | 2 |
|  | T3/T4 | 0 |
| Histological sub-type | Clear cell type | 18 |
|  | Chromophobe type | 2 |
|  | Papillary type | 1 |
|  | Other types | 0 |
