# Supplementary material for: Fast quantitative urinary proteomic profiling workflow for biomarker discovery in kidney cancer
Source: Clin Proteomics. 2018 Dec 22;15:42. doi: 10.1186/s12014-018-9220-2 (PMC6303996; doi:10.1186/s12014-018-9220-2)
Supplement: Supplementary file 2 — Additional file 2: Table S2. A summary of the vDIA method. [file 12014_2018_9220_MOESM2_ESM.docx]

**Table S2.** A summary of the vDIA method.

| **LC gradient:** | 80 min | | | |
| --- | --- | --- | --- | --- |
| **MS Method :** | One full scan and 30 variable window DIA scans | | | |
| **Window List:** | | | | |
| Window No | start_mz | end_mz | center | Width |
| 1 | 349 | 374 | 361.5 | 25 |
| 2 | 374 | 393 | 383.5 | 19 |
| 3 | 393 | 408 | 400.5 | 15 |
| 4 | 408 | 420 | 414 | 12 |
| 5 | 420 | 432 | 426 | 12 |
| 6 | 432 | 442 | 437 | 10 |
| 7 | 442 | 454 | 448 | 12 |
| 8 | 454 | 465 | 459.5 | 11 |
| 9 | 465 | 477 | 471 | 12 |
| 10 | 477 | 488 | 482.5 | 11 |
| 11 | 488 | 499 | 493.5 | 11 |
| 12 | 499 | 509 | 504 | 10 |
| 13 | 509 | 521 | 515 | 12 |
| 14 | 521 | 532 | 526.5 | 11 |
| 15 | 532 | 545 | 538.5 | 13 |
| 16 | 545 | 558 | 551.5 | 13 |
| 17 | 558 | 571 | 564.5 | 13 |
| 18 | 571 | 589 | 580 | 18 |
| 19 | 589 | 604 | 596.5 | 15 |
| 20 | 604 | 620 | 612 | 16 |
| 21 | 620 | 638 | 629 | 18 |
| 22 | 638 | 659 | 648.5 | 21 |
| 23 | 659 | 679 | 669 | 20 |
| 24 | 679 | 701 | 690 | 22 |
| 25 | 701 | 730 | 715.5 | 29 |
| 26 | 730 | 768 | 749 | 38 |
| 27 | 768 | 810 | 789 | 42 |
| 28 | 810 | 857 | 833.5 | 47 |
| 29 | 857 | 947 | 902 | 90 |
| 30 | 947 | 1489 | 1218 | 542 |
