# Supplementary material for: Fast quantitative urinary proteomic profiling workflow for biomarker discovery in kidney cancer
Source: Clin Proteomics. 2018 Dec 22;15:42. doi: 10.1186/s12014-018-9220-2 (PMC6303996; doi:10.1186/s12014-018-9220-2)
Supplement: Supplementary file 3 — Additional file 3: Table S3. A summary of the classic DIA method. [file 12014_2018_9220_MOESM3_ESM.docx]

**Table S3.** A summary of the classic DIA method.

| **LC gradient:** | 80 min | | | |
| --- | --- | --- | --- | --- |
| **MS Method :** | One full scan and 32 fixed window DIA scans | | | |
| **Window List:** | | | | |
| Window No | start_mz | end_mz | center | Width |
| 1 | 399.9376 | 425.9376 | 412.9376 | 26 |
| 2 | 424.9490 | 450.9490 | 437.9490 | 26 |
| 3 | 449.9603 | 475.9603 | 462.9603 | 26 |
| 4 | 474.9717 | 500.9717 | 487.9717 | 26 |
| 5 | 499.9831 | 525.9831 | 512.9831 | 26 |
| 6 | 524.9944 | 550.9944 | 537.9944 | 26 |
| 7 | 550.0058 | 576.0058 | 563.0058 | 26 |
| 8 | 575.0172 | 601.0172 | 588.0172 | 26 |
| 9 | 600.0285 | 626.0285 | 613.0285 | 26 |
| 10 | 625.0399 | 651.0399 | 638.0399 | 26 |
| 11 | 650.0513 | 676.0513 | 663.0513 | 26 |
| 12 | 675.0626 | 701.0626 | 688.0626 | 26 |
| 13 | 700.0740 | 726.0740 | 713.0740 | 26 |
| 14 | 725.0854 | 751.0854 | 738.0854 | 26 |
| 15 | 750.0967 | 776.0967 | 763.0967 | 26 |
| 16 | 775.1081 | 801.1081 | 788.1081 | 26 |
| 17 | 800.1195 | 826.1195 | 813.1195 | 26 |
| 18 | 825.1309 | 851.1309 | 838.1309 | 26 |
| 19 | 850.1422 | 876.1422 | 863.1422 | 26 |
| 20 | 875.1536 | 901.1536 | 888.1536 | 26 |
| 21 | 900.1650 | 926.1650 | 913.1650 | 26 |
| 22 | 925.1763 | 951.1763 | 938.1763 | 26 |
| 23 | 950.1877 | 976.1877 | 963.1877 | 26 |
| 24 | 975.1991 | 1001.1991 | 988.1991 | 26 |
| 25 | 1000.2104 | 1026.2104 | 1013.2104 | 26 |
| 26 | 1025.2218 | 1051.2218 | 1038.2218 | 26 |
| 27 | 1050.2332 | 1076.2332 | 1063.2332 | 26 |
| 28 | 1075.2445 | 1101.2445 | 1088.2445 | 26 |
| 29 | 1100.2559 | 1126.2559 | 1113.2559 | 26 |
| 30 | 1125.2673 | 1151.2673 | 1138.2673 | 26 |
| 31 | 1150.2786 | 1176.2786 | 1163.2786 | 26 |
| 32 | 1175.2900 | 1201.2900 | 1188.2900 | 26 |
