# Supplementary material for: Fast quantitative urinary proteomic profiling workflow for biomarker discovery in kidney cancer
Source: Clin Proteomics. 2018 Dec 22;15:42. doi: 10.1186/s12014-018-9220-2 (PMC6303996; doi:10.1186/s12014-018-9220-2)
Supplement: Supplementary file 4 — Additional file 4: Table S4. Digestion efficiency of the SISPROT technology in processing urine samples. [file 12014_2018_9220_MOESM4_ESM.docx]

**Table S4.** Digestion efficiency of the SISPROT technology in processing urine samples.

| Sample | PSM (s) | **PSM-Missed ≤ 2 (%)** | PSM-Missed = 0 (%) | PSM-Missed = 1 (%) | PSM-Missed = 2 (%) | PSM-Missed = 3(%) | PSM-Missed = 4(%) | PSM-Missed = 5(%) |
| --- | --- | --- | --- | --- | --- | --- | --- | --- |
| DDA-1 | 4417 | **98.78** | 77.11 | 18.07 | 3.60 | 0.97 | 0.20 | 0.05 |
| DDA-2 | 4604 | **98.91** | 76.93 | 18.31 | 3.67 | 0.89 | 0.13 | 0.07 |
| DDA-3 | 4590 | **98.96** | 77.26 | 18.10 | 3.60 | 0.89 | 0.11 | 0.04 |
