# Supplementary material for: Fast quantitative urinary proteomic profiling workflow for biomarker discovery in kidney cancer
Source: Clin Proteomics. 2018 Dec 22;15:42. doi: 10.1186/s12014-018-9220-2 (PMC6303996; doi:10.1186/s12014-018-9220-2)
Supplement: Supplementary file 5 — Additional file 5: Table S5. Overview of the spectral libraries. [file 12014_2018_9220_MOESM5_ESM.docx]

**Table S5.** Overview of the spectral libraries.

| Library | Protein groups | Proteins | Peptides | Fragment ions | Instrument | Source |
| --- | --- | --- | --- | --- | --- | --- |
| 1 | 1256 | 2255 | 7913 | 60784 | Orbitrap Fusion | Project specific library |
| 2 | 1765 | 3009 | 11166 | 77055 | Q Exactive HF | Online urine reference library |
| 3 | 10524 | 10643 | 149066 | 1252796 | TripleTOF 5600+ | Pan Human library |
