# Supplementary material for: Fast quantitative urinary proteomic profiling workflow for biomarker discovery in kidney cancer
Source: Clin Proteomics. 2018 Dec 22;15:42. doi: 10.1186/s12014-018-9220-2 (PMC6303996; doi:10.1186/s12014-018-9220-2)
Supplement: Supplementary file 6 — Additional file 6: Figure S1. Correlation of the quantified protein intensities between (a) replicate 1 and 2, and (b) replicate 2 and 3. [file 12014_2018_9220_MOESM6_ESM.docx]

**
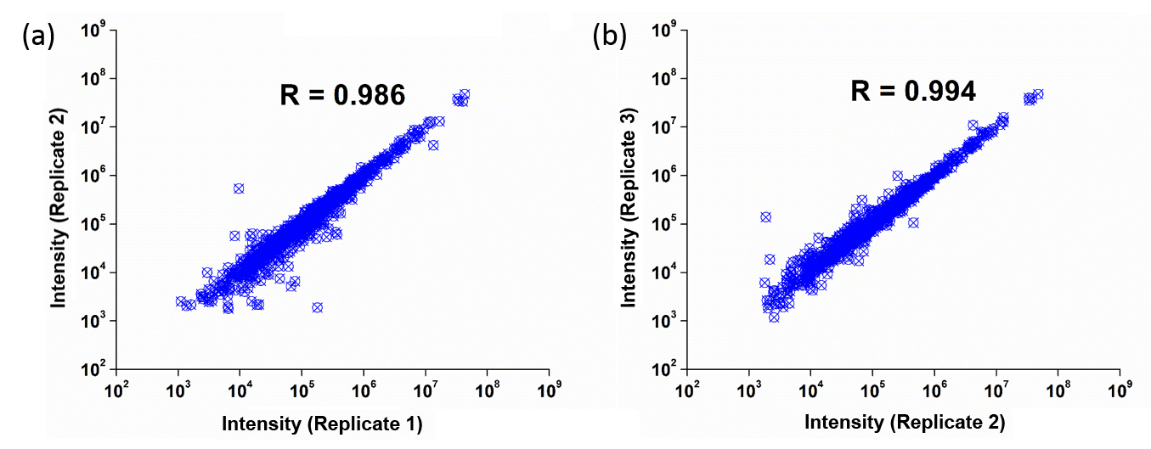
**

**Figure S1.** Correlation of the quantified protein intensities between (a) replicate 1 and 2, and (b) replicate 2 and 3.
