# Supplementary material for: Fast quantitative urinary proteomic profiling workflow for biomarker discovery in kidney cancer
Source: Clin Proteomics. 2018 Dec 22;15:42. doi: 10.1186/s12014-018-9220-2 (PMC6303996; doi:10.1186/s12014-018-9220-2)
Supplement: Supplementary file 7 — Additional file 7: Figure S2. Performance of the urinary proteome profiling workflow in peptide level. (a) Peptide identification reproducibility of the entire workflow. (b–d) Correlation of the peptide intensities between individual replicates. [file 12014_2018_9220_MOESM7_ESM.docx]

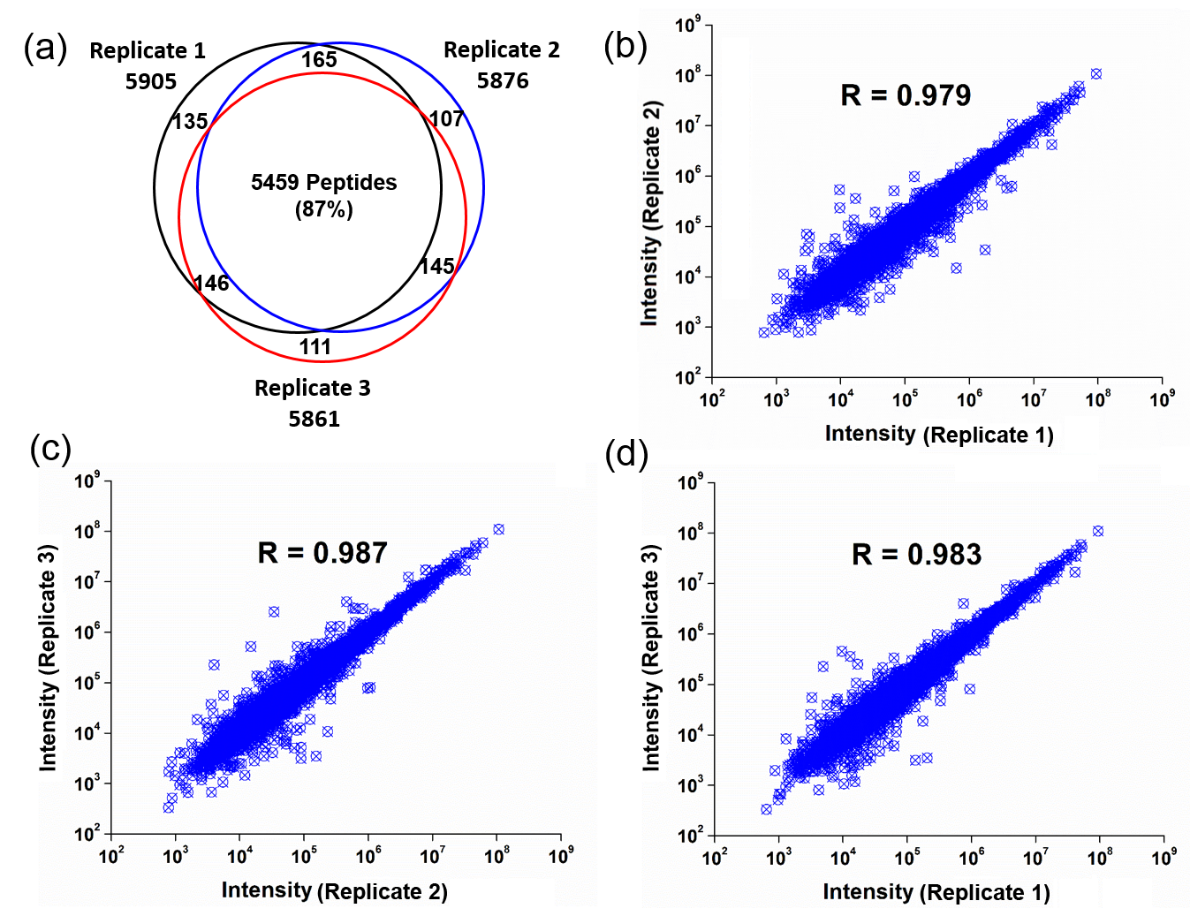


**Figure S2.** Performance of the urinary proteome profiling workflow in peptide level. (a) Peptide identification reproducibility of the entire workflow. (b−d) Correlation of the peptide intensities between individual replicates.
