# Supplementary material for: Fast quantitative urinary proteomic profiling workflow for biomarker discovery in kidney cancer
Source: Clin Proteomics. 2018 Dec 22;15:42. doi: 10.1186/s12014-018-9220-2 (PMC6303996; doi:10.1186/s12014-018-9220-2)
Supplement: Supplementary file 9 — Additional file 9: Table S7. Significantly changed proteins found in KC patients. [file 12014_2018_9220_MOESM9_ESM.docx]

**Table S7.** Significantly changed proteins found in KC patients.

| Uniprot ID | Protein Name | p-value^a^ | Fold change^b^ |
| --- | --- | --- | --- |
| O94910 | Adhesion G protein-coupled receptor L1 | 1.53E-06 | -4.2 |
| Q9H8L6 | Multimerin-2 | 1.61E-05 | -2.3 |
| O15197 | Ephrin type-B receptor 6 | 4.34E-05 | -1.6 |
| P16112 | Aggrecan core protein | 5.49E-05 | -2.5 |
| P01210 | Proenkephalin-A | 1.02E-04 | -1.9 |
| Q96GW7 | Brevican core protein | 1.03E-04 | -1.8 |
| Q6UY11 | Protein delta homolog 2 | 1.58E-04 | -1.6 |
| Q6UVK1 | Chondroitin sulfate proteoglycan 4 | 2.20E-04 | -1.5 |
| Q9H461 | Frizzled-8 | 2.93E-04 | -1.5 |
| Q96A25 | Transmembrane protein 106A | 3.75E-04 | -2.9 |
| P08572 | Collagen alpha-2(IV) chain | 4.09E-04 | -1.6 |
| P05090 | Apolipoprotein D | 4.60E-04 | -1.7 |
| Q8IYJ0 | PILR alpha-associated neural protein | 8.05E-04 | -3.3 |
| Q92673 | Sortilin-related receptor | 8.68E-04 | -1.6 |
| Q6EMK4 | Vasorin | 1.05E-03 | -1.6 |
| O75339 | Cartilage intermediate layer protein 1 | 1.12E-03 | -1.8 |
| Q9UIB8 | SLAM family member 5 | 1.14E-03 | -1.7 |
| O94772 | Lymphocyte antigen 6H | 1.14E-03 | -1.8 |
| Q96IU4 | Protein ABHD14B | 1.27E-03 | -1.9 |
| P05154 | Plasma serine protease inhibitor | 1.27E-03 | -1.7 |
| Q15517 | Corneodesmosin | 1.38E-03 | -2.1 |
| P11117 | Lysosomal acid phosphatase | 1.48E-03 | -1.6 |
| Q9ULI3 | Protein HEG homolog 1 | 1.56E-03 | -1.8 |
| P01189 | Pro-opiomelanocortin | 1.67E-03 | -2.9 |
| Q8IUL8 | Cartilage intermediate layer protein 2 | 1.74E-03 | -1.9 |
| P61106 | Ras-related protein Rab-14 | 1.88E-03 | -1.5 |
| P82980 | Retinol-binding protein 5 | 1.95E-03 | -1.8 |
| Q7Z3B1 | Neuronal growth regulator 1 | 2.15E-03 | -1.8 |
| O43155 | Leucine-rich repeat transmembrane protein FLRT2 | 2.31E-03 | -1.6 |
| Q7Z4W1 | L-xylulose reductase | 2.36E-03 | -2.2 |
| Q14894 | Ketimine reductase mu-crystallin | 2.38E-03 | -2 |
| O95865 | N(G),N(G)-dimethylarginine dimethylaminohydrolase 2 | 2.48E-03 | -1.6 |
| P08582 | Melanotransferrin | 2.54E-03 | -1.6 |
| B4E1Z4 | cDNA FLJ55673, highly similar to Complement factor B (EC 3.4.21.47) | 2.56E-03 | 4.3 |
| Q96DR8 | Mucin-like protein 1 | 2.60E-03 | -2.5 |
| Q14982 | Opioid-binding protein/cell adhesion molecule | 3.42E-03 | -1.6 |
| P05452 | Tetranectin | 3.65E-03 | -1.8 |
| P12273 | Prolactin-inducible protein | 4.71E-03 | -3.2 |
| P01768 | Immunoglobulin heavy variable 3-30 | 4.93E-03 | 1.6 |
| P04062 | Glucosylceramidase | 5.05E-03 | -2.1 |
| Q07507 | Dermatopontin | 5.11E-03 | -1.6 |
| Q08174 | Protocadherin-1 | 5.65E-03 | -1.7 |
| P00338 | L-lactate dehydrogenase A chain | 5.68E-03 | -1.5 |
| P31146 | Coronin-1A | 6.12E-03 | 25.7 |
| P16152 | Carbonyl reductase [NADPH] 1 | 6.30E-03 | -1.8 |
| Q92563 | Testican-2 | 6.52E-03 | -1.6 |
| P06576 | ATP synthase subunit beta, mitochondrial | 6.67E-03 | 2.7 |
| Q5KU26 | Collectin-12 | 6.84E-03 | -2.5 |
| E2QRG1 | Phospholipase D3 | 6.91E-03 | -2.1 |
| P48061 | Stromal cell-derived factor 1 | 7.10E-03 | -2 |
| O75074 | Low-density lipoprotein receptor-related protein 3 | 7.45E-03 | 6.5 |
| Q9P121 | Neurotrimin | 7.47E-03 | -1.8 |
| P25325 | 3-mercaptopyruvate sulfurtransferase | 8.30E-03 | -1.8 |
| O60635 | Tetraspanin-1 | 8.41E-03 | -1.9 |
| Q86YD3 | Transmembrane protein 25 | 8.59E-03 | -2.1 |
| P55259 | Pancreatic secretory granule membrane major glycoprotein GP2 | 8.96E-03 | -2.1 |
| P23470 | Receptor-type tyrosine-protein phosphatase gamma | 9.03E-03 | -1.6 |
| P01782 | Immunoglobulin heavy variable 3-9 | 9.03E-03 | 1.7 |
| P01705 | Immunoglobulin lambda variable 2-23 | 9.28E-03 | 2.4 |
| P08962 | CD63 antigen | 9.96E-03 | -1.5 |
| Q96DA0 | Zymogen granule protein 16 homolog B | 9.96E-03 | -1.5 |
| P55000 | Secreted Ly-6/uPAR-related protein 1 | 1.01E-02 | -1.7 |
| P13598 | Intercellular adhesion molecule 2 | 1.02E-02 | -1.8 |
| P11597 | Cholesteryl ester transfer protein | 1.09E-02 | -1.6 |
| P04054 | Phospholipase A2 | 1.28E-02 | 1.6 |
| Q96RW7 | Hemicentin-1 | 1.31E-02 | -1.8 |
| Q7Z7M0 | Multiple epidermal growth factor-like domains protein 8 | 1.31E-02 | -2.9 |
| Q99574 | Neuroserpin | 1.33E-02 | -1.6 |
| P36871 | Phosphoglucomutase-1 | 1.45E-02 | -5.7 |
| A0A0B4J1V0 | Immunoglobulin heavy variable 3-15 | 1.58E-02 | 1.9 |
| A0A0C4DH68 | Immunoglobulin kappa variable 2-24 | 1.64E-02 | 2 |
| Q9HBJ8 | Collectrin | 1.65E-02 | -2 |
| Q9Y653 | Adhesion G-protein coupled receptor G1 | 1.81E-02 | -1.6 |
| P00746 | Complement factor D | 1.81E-02 | 2.3 |
| P35318 | ADM | 1.82E-02 | -1.9 |
| P04733 | Metallothionein-1F | 1.82E-02 | 2.5 |
| Q8NFZ8 | Cell adhesion molecule 4 | 1.85E-02 | -1.5 |
| A0A0A0MS15 | Immunoglobulin heavy variable 3-49 | 1.88E-02 | 3 |
| Q9UHX3 | Adhesion G protein-coupled receptor E2 | 1.89E-02 | -2.9 |
| P22891 | Vitamin K-dependent protein Z | 2.11E-02 | -1.6 |
| P01876 | Ig alpha-1 chain C region | 2.24E-02 | 1.7 |
| Q9Y6W3 | Calpain-7 | 2.25E-02 | -2.2 |
| [P00749](http://www.uniprot.org/uniprot/P00749) | Urokinase-type plasminogen activator | 2.26E-02 | -1.6 |
| P34896 | Serine hydroxymethyltransferase, cytosolic | 2.29E-02 | -1.7 |
| P01857 | Ig gamma-1 chain C region | 2.32E-02 | 1.6 |
| P41181 | Aquaporin-2 | 2.34E-02 | -2.2 |
| Q8TBZ0 | Coiled-coil domain-containing protein 110 | 2.37E-02 | 6.5 |
| Q14393 | Growth arrest-specific protein 6 | 2.40E-02 | -1.8 |
| P09651 | Heterogeneous nuclear ribonucleoprotein A1 | 2.40E-02 | 3.3 |
| P62805 | Histone H4 | 2.59E-02 | 2.2 |
| Q5D862 | Filaggrin-2 | 2.63E-02 | 3 |
| Q96S96 | Phosphatidylethanolamine-binding protein 4 | 2.65E-02 | -2.2 |
| Q03403 | Trefoil factor 2 | 2.70E-02 | -1.5 |
| Q6UX73 | UPF0764 protein C16orf89 | 2.75E-02 | -2 |
| P99999 | Cytochrome c | 2.95E-02 | 2.6 |
| O14798 | Tumor necrosis factor receptor superfamily member 10C | 3.00E-02 | -1.9 |
| P02792 | Ferritin light chain | 3.04E-02 | 2 |
| P35558 | Phosphoenolpyruvate carboxykinase, cytosolic [GTP] | 3.05E-02 | -1.9 |
| P02794 | Ferritin heavy chain | 3.09E-02 | 1.7 |
| B9A064 | Immunoglobulin lambda-like polypeptide 5 | 3.15E-02 | 2.4 |
| Q9NVN3 | Synembryn-B | 3.15E-02 | 9.8 |
| P19652 | Alpha-1-acid glycoprotein 2 | 3.16E-02 | 1.9 |
| P08195 | 4F2 cell-surface antigen heavy chain | 3.39E-02 | -1.5 |
| P22626 | Heterogeneous nuclear ribonucleoproteins A2/B1 | 3.45E-02 | 14.7 |
| A0A0B4J1V2 | Immunoglobulin heavy variable 2-26 | 3.67E-02 | 2.7 |
| P29279 | Connective tissue growth factor | 3.73E-02 | -1.5 |
| P20160 | Azurocidin | 3.84E-02 | 8.6 |
| P52823 | Stanniocalcin-1 | 3.84E-02 | -1.7 |
| P09486 | SPARC | 3.88E-02 | -3.2 |
| O60814 | Histone H2B type 1-K | 3.91E-02 | 2.3 |
| A0A0J9YX35 | Immunoglobulin heavy variable 3-64D | 3.93E-02 | 1.7 |
| Q9UHG2 | ProSAAS | 4.00E-02 | -2 |
| P52566 | Rho GDP-dissociation inhibitor 2 | 4.00E-02 | 4.2 |
| P02656 | Apolipoprotein C-III | 4.02E-02 | 3.4 |
| Q9UHL4 | Dipeptidyl peptidase 2 | 4.11E-02 | -1.6 |
| P11362 | Fibroblast growth factor receptor 1 | 4.20E-02 | -1.6 |
| P16401 | Histone H1.5 | 4.30E-02 | 4.6 |
| Q13387 | C-Jun-amino-terminal kinase-interacting protein 2 | 4.32E-02 | 1.6 |
| P13646 | Cytokeratin-13 | 4.38E-02 | 2.2 |
| P12814 | Alpha-actinin-1 | 4.40E-02 | 3.2 |
| C9IYI1 | Dermokine | 4.79E-02 | -1.7 |
| P00736 | Complement C1r subcomponent | 4.80E-02 | 5.7 |
| Q9H299 | SH3 domain-binding glutamic acid-rich-like protein 3 | 4.85E-02 | 2 |
| P22894 | Neutrophil collagenase | 4.86E-02 | 3.5 |
| P04211 | Immunoglobulin lambda variable 7-43 | 4.94E-02 | 1.7 |
| ^a^The p value was calculated using Student’s t-test. ^b^ Fold change with positive value demonstrates up-regulated expression in KC patients, and negative value indicates down-regulated expression compared to controls. | | | |
